# SANReSP: A new Italian questionnaire to screen patients for obstructive sleep apnea

**DOI:** 10.1371/journal.pone.0276217

**Published:** 2022-10-14

**Authors:** Salvatore Romano, Anna Lo Bue, Adriana Salvaggio, Luis V. F. Oliveira, Luigi Ferini-Strambi, Giuseppe Insalaco

**Affiliations:** 1 National Research Council of Italy, Institute for Biomedical Research and Innovation (IRIB), Palermo, Italy; 2 National Research Council of Italy, Institute of Translational Pharmacology (IFT), Palermo, Italy; 3 Evangelical University of Goiás (UniEVANGÉLICA), Postgraduate Program in Human Movement and Rehabilitation–PPGMHR, Anapolis, Brazil; 4 Università Vita-Salute San Raffaele, and Division of Neuroscience, IRCCS San Raffaele Scientific Institute, Milan, Italy; University of Rome Tor Vergata, ITALY

## Abstract

**Purpose:**

Obstructive sleep apnea (OSA) is a common, prevalent, and underdiagnosed disorder. Its lack of diagnosis and treatment is associated with increased morbidity and mortality. Previous screening questionnaires investigated parameters including body mass index, age, neck circumference, and sex, in addition to symptoms. This study aimed to validate a new Italian, self-administered, and easy-to-use six-item questionnaire that evaluates only subjective symptoms.

**Patients and methods:**

The present study included 2622 patients (male, 2011; female, 611). Patients who were at least 18 years old, spoke Italian, referred to our sleep clinic for possible OSA, and completed the self-administered SANReSP questionnaire were recruited for the study. The predictive performance of the questionnaire was also evaluated.

**Results:**

Nocturnal study showed 89.9% of OSA patients had apnea-hypopnea index (AHI) ≥ 5/h; 68.7%, AHI ≥ 15/h; and 48.2%, AHI ≥ 30/h. The optimal SANReSP score for AHI ≥ 5/h was >3 with a sensitivity and specificity of 74.76% and 67.92%, respectively, and an area under receiver operating characteristic curve (ROC) of 0.76. For moderate–severe OSA, the optimal SANReSP score was >3 (sensitivity, 78.18%; specificity, 46.53%; ROC, 0.66). For severe OSA, the optimal SANReSP score was >4 (sensitivity, 59.10%; specificity, 64.73%; ROC, 0.65). The probability of OSA increased with higher SANReSP scores (98.7% and 97.9% in men and women, respectively).

**Conclusion:**

The SANReSP questionnaire is a short, easy-to-use, and self-administered screening tool for OSA. Its performance is similar to that of other widely used questionnaires; furthermore, it is advantageous in that it does not require anthropometric measurements.

## Introduction

Obstructive sleep apnea (OSA) is characterised by recurrent episodes of partial or complete obstruction of the upper respiratory tract during sleep. It has been widely demonstrated that OSA is accompanied by a higher frequency of road, domestic, and workplace accidents and a decline in neurocognitive and psychosocial functions, which can have significant repercussions on quality of life [1]. Sleep apnea affects normal sleep architecture and causes intermittent hypoxaemia, oxidative stress, and increased sympathetic tone, resulting in cardiovascular, cerebrovascular, metabolic, and neurocognitive sequelae. Risk factors include obesity as well as soft and skeletal facial tissue abnormalities [2, 3].

OSA presents with daytime symptoms such as fatigue, unrefreshing sleep, and excessive sleepiness and nocturnal symptoms such as persistent and intermittent snoring, sleep apnea reported by the bed partner, and nocturia, especially in younger individuals without any urological disorders. The prevalence of nocturia in patients with OSA is 50–70% [4]. Women with OSA often exhibit atypical symptoms, such as insomnia, mood changes, and headache [5].

Substantial methodological heterogeneity in population prevalence studies cause a wide variation in the reported prevalence, which, in general, is high. At ≥5 events/h apnea-hypopnea index (AHI), the overall population prevalence range from 9% to 38% and as higher in men. At ≥15 events/h AHI, the prevalence in the general adult population range from 6% to 17%, being as high as 49% in the advanced ages [6].

A recent epidemiological study, using AHI ≥ 5/h as the criterion, estimates that nearly 1 billion (936 million) adults aged 30–69 years could have OSA, out of which the number of individuals with moderate–severe OSA (AHI ≥ 15/h), for which treatment is generally recommended, is estimated to be nearly 425 million [7].

Given the high prevalence and impact of OSA on health and quality of life, it is necessary to have screening tools for identifying OSA and preventing related comorbidities as well as health, social, and economic implications. To date, insufficient sensitive and specific criteria necessitate the clinical diagnosis of OSA. Nocturnal laboratory or home-based polysomnography and cardio-respiratory monitoring are the only diagnostic procedures. However, these methods are expensive in terms of economic and organisational resources, and they are not always immediately available. Thus, most people with moderate–severe OSA are not diagnosed [8].

Several assessment tools have been developed to expedite the identification of patients at risk for OSA. The different screening tools, mainly characterised by questionnaires, are generally based on the main clinical and anthropometric risk factors for OSA. Comparison between the different questionnaires can be difficult due to the heterogeneity of the studies, including the target population chosen and the different AHI cut-offs and type of examination instruments used for OSA diagnosis. The Berlin questionnaire (BQ), created in the field of general medicine, and the STOP-BANG questionnaire (SBQ), developed in the anaesthesiology field, are widely used for OSA diagnosis, but their predictivity vary depending on the area in which they are administered [9].

Given that OSA diagnosis using sleep studies is costly and time-consuming, and the existing screening tools have a significant tradeoff between sensitivity and specificity, there is a need to develop new tools that perform better and has no need of physical measures which are inherent disadvantages of the existing tools. We therefore thought is useful the development of an OSA screening tool based solely on symptoms.

In our study, we developed a self-administered six-item screening questionnaire for OSA with dichotomous answers, without the need of anthropometric measurements, called SANReSP.

## Material and methods

### Participants

The study involved Italian patients consulting our sleep center for respiratory sleep disorders. This study was conducted in accordance with the amended Declaration of Helsinki. The Institutional Ethical Committee, Azienda Ospedaliera Universitaria Policlinico “Paolo Giaccone”, Palermo 1, approved the protocol. All patients gave their written informed consent for personal data processing.

Recruitment was conducted between January 2015 and June 2019. The study recruited 2622 patients (male, 2011; female, 611). Italian-speaking patients referred to our sleep clinic because of OSA suspicion and at least 18 years of age were eligible for the study. Patients with uncontrolled psychiatric illness, neurocognitive impairment, usage of sedative or hypnotic medications, sleep time test duration shorter than 6 h, or “absence of answer” answer to at least one of the SANReSP items were not included in the study. The minimum sample size required for a screening study, with the null hypothesis for sensitivity and specificity at 0.5, a target significance level of 0.05, and a power of 80%, ranges from 22 (prevalence = 90%) to 980 (prevalence = 5%) [10]. The prevalence of OSA in patients at a sleep clinic is estimated to be approximately 80%. Thus, the study’s sample size of 2622 patients was consistent.

### Sample processing

A portable, computerised system type III (Embla, Natus Inc., Middleton, USA) was employed to perform nocturnal monitoring. The sleep studies were performed at the sleep lab and at patients’ home. A sleep technician has set sleep studies device. The following signals were recorded: airflow by nasal cannula pressure, snoring, thoracic and abdominal efforts, body position, limb movements, arterial oxygen saturation, pulse rate, and pulse waveform. The recording duration was at least 6 h [11].

Type III Sleep Studies uses portable monitors that allow sleep studies to be done at the patient’s home or elsewhere. This option was introduced as a more accessible and less expensive alternative to in-laboratory polysomnography. Unlike Type I, Type III testing cannot measure the duration of sleep, the number of arousals or sleep stages, nor can it detect non respiratory sleep disorders. The American Academy of Sleep Medicine and Canadian Sleep Society/Canadian Thoracic Society guidelines recommend that portable sleep studies be provided under the direction of health professionals with accreditation in sleep medicine and as part of a comprehensive assessment [11].

Apneas and hypopneas were visually scored and OSA severity was defined according to the American Academy of Sleep Medicine 2012 standard criteria [12]. Apneas were identified on the airflow signal and were classified as obstructive, central, or mixed according to the behavior of thoraco-abdominal movements. Hypopneas were defined as discernible reductions in airflow or thoraco-abdominal movements for at least 10 s, followed by an arterial oxygen saturation fall of ≥3%. The AHI was calculated as the number of apneas and hypopneas per hour of estimated total sleep time. The percentage of the night with O_2_ saturation <90% (TSat_90_) was assessed. A sleep medicine expert, blinded to the questionnaire, scored all sleep studies.

### Questionnaire

**Fig 1:**
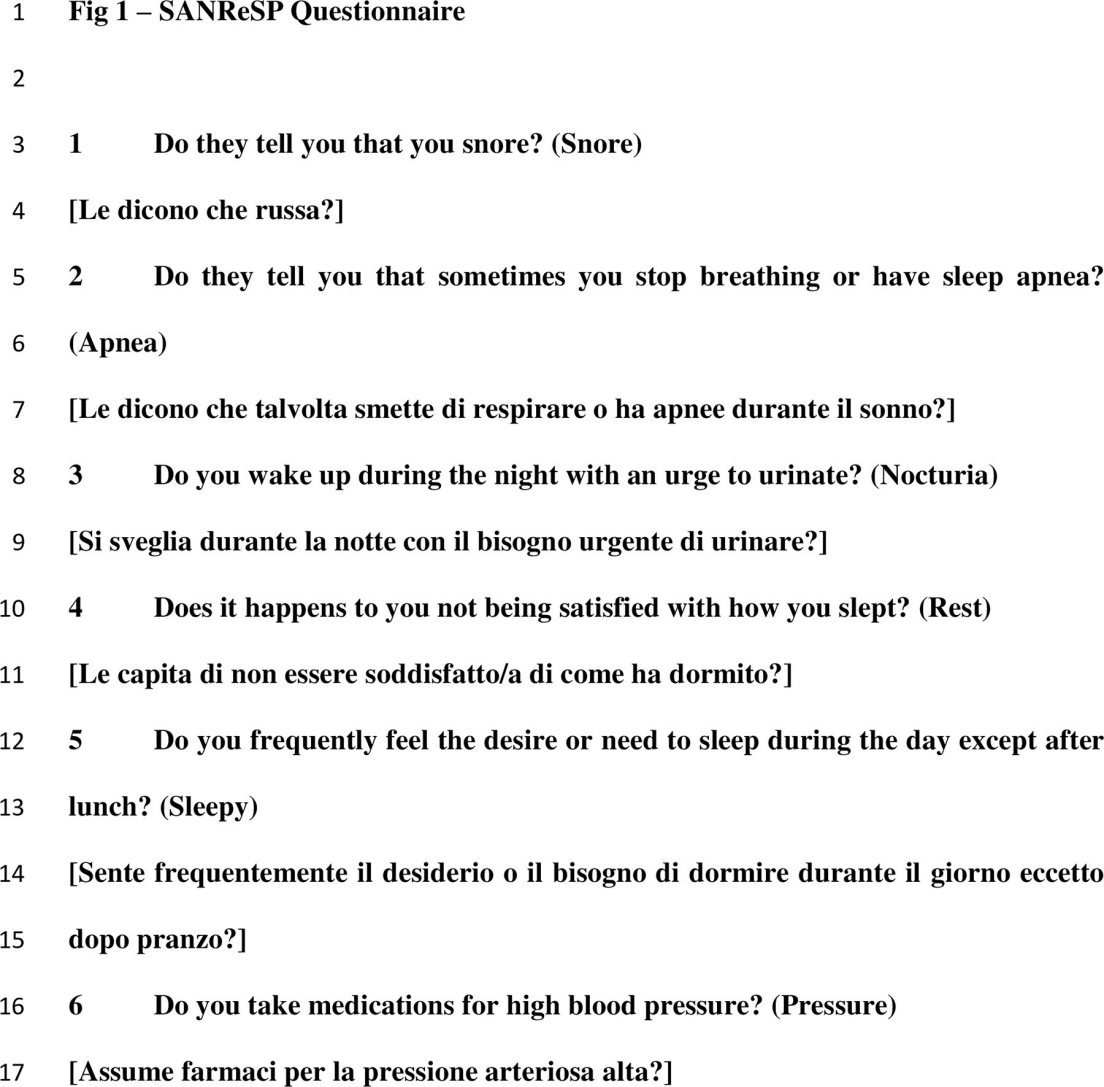
SANReSP questionnaire.

To keep the questionnaire concise, easy-to-use, and quick to fill out, the questions were designed in a yes/no format. Based on a literature review, questions were designed using the most common clinical domains of OSA and hypertension. The SANReSP questionnaire includes six dichotomous (yes/no) questions: **S:** Do they tell you that you **S**nore? [Le dicono che russa?], **A:** Do they tell you that sometimes you stop breathing or have sleep **A**pnea? [Le dicono che talvolta smette di respirare o ha apnee durante il sonno?], **N:** Do you wake up during the night with an urge to urinate ‘**N**octuria’? [Si sveglia durante la notte con il bisogno urgente di urinare?], **Re:** Do you sometimes feel unsatisfied with how you slept ‘**Re**st’? [Le capita di non essere soddisfatto/a di come ha dormito?], **S:** Do you frequently feel the desire or need to sleep during the day except after lunch ‘**S**leepy’? [Sente frequentemente il desiderio o il bisogno di dormire durante il giorno eccetto dopo pranzo?], and **P:** Do you take medications for high blood **P**ressure? [Assume farmaci per la pressione arteriosa alta?] (Fig 1). SANReSP is a self-administered questionnaire. The score ranges from 0 to 6, attributing one point for each affirmative response and 0 is assigned when no answer is given.

### Statistical analyses

Statistical analyses were performed using the commercial 2020 MedCalc statistical software (MedCalc Software Ltd., Ostend, Belgium). The Kolmogorov–Smirnov test was used for evaluating the accordance of the parameters with a normal distribution. Normally distributed continuous variables were reported as means and standard deviations. Non-normally distributed continuous variables were reported as median and 25–75% interquartile range. Categorical variables were reported as frequencies. The non-parametric Wilcoxon’s test was used to assess the differences. Comparisons with a p-value of ≤0.05 were considered statistically significant.

The OSA probability determined by SANReSP was compared to the diagnostic sleep study results. Receiver operating characteristic (ROC) curve analysis was performed. The specificity, sensitivity, positive predictive value (PPV), negative predictive value (NPV), likelihood ratio (LR) for a positive test result (LR+), LR for a negative test result (LR-), accuracy, and odds ratio (LR+/LR-) (OR) were calculated. All estimates were reported with their respective 95% confidence intervals (CIs). The CIs for sensitivity, specificity, and accuracy were ‘exact’ Clopper–Pearson CIs. The CIs for the LRs was calculated using the ‘Log method’ of Altman et al. 2000 (as given on page 109) [13]. The CIs for the predictive values were the standard logit CIs given by Mercaldo et al. 2007 [14]. The CIs for OR were calculated according to Altman DG 1991 [15].

The LR+ and LR- indicate if a patient is more or less likely to have a disease, respectively. Since LRs are not affected by disease prevalence, they might be more useful parameters than sensitivity, specificity, and predictive values. The higher the LR+ (>1), the stronger is the prediction of a disease. Conversely, the lower the LR- (<0.5), the better is the prediction of the absence of disease.

The optimal cut-off that maximises true positives and minimises false positives was determined, and it corresponds to the highest sensitivity + specificity. The response was dichotomised by AHI ≥ 5/h for all OSA patients, AHI ≥ 15/h for moderate–severe OSA patients, and AHI ≥ 30/h for only severe OSA patients. The predictive probability (post-test probability) was calculated using logistic regression. The statistical analyses were conducted separately for men and women. The statistical significance of the difference between the areas under ROC curves (AUCs) of men and women was evaluated using the DeLong method [16].

## Results

### Demographic characteristics

Descriptive statistics of the analysed sample are presented in Table 1. Body mass index (BMI) and age were greater in women than in men, while neck circumference and AHI severity were greater in men than in women. Of the 2622 patients, 265 (10.1%) patients did not have OSA, 2357 (89.9%) had AHI ≥ 5/h, 1801 (68.7%) had AHI ≥ 15/h, and 1264 (48.2%) had AHI ≥ 30/h.

**Table 1:** Characteristics of the population.

Parameter	All	Males	Females
N	2622	2011	611
BMI (kg/m^2^)	31.2 (27.6–35.9)	30.9 (27.5–35.0)	32.8 (27.7–38.6)*
Age (y)	56 (47–64)	55 (46–63)	59 (51–68) *
Neck size (cm)	41 (39–77)	42 (40–44)	37 (35–40) *
AHI (n/h)	28 (11–55)	30 (13–57)	19 (8–45) *
AHI< 5 n/h (%)	265 (10.1)	171 (8.5)	94 (15.3)
AHI≥ 5 n/h (%)	2357 (89.9)	1840 (91.5)	517 (84.6)
AHI≥ 15 n/h (%)	1801 (68.7)	1441 (71.6)	360 (58.9)
AHI≥ 30 n/h (%)	1264 (48.2)	1037 (51.6)	227 (37.1)
SANReSP Score	4 (3–5)	4 (3–5)	5 (4–5)
Hypertension	1319 (50.3)	973 (48.4)	346 (56.6)
Atrial fibrillation	115 (4.4)	92 (4.6)	23 (3.7)
Heart failure	120 (4.6)	90 (4.5)	30 (4.9)
Coronary heart disease	168 (6.4)	152 (7.5)	16 (2.6)
Cerebrovascular diseases	111 (4.2)	78 (3.9)	33 (5.4)
Lung disease	234 (8.9)	164 (8.1)	70 (11.5)
Diabetes	325 (12.4)	229 (11.4)	96 (15.7)

Data are presented as median (25% - 75% interquartile range) or number (percentage). BMI = body mass index; AHI = apnea-hypopnea index. Non-parametric Wilcoxon’s test was used to compared each variable of Males vs Females.

* = *p* < 0.0001.

### Questionnaire analysis

**Fig 2:**
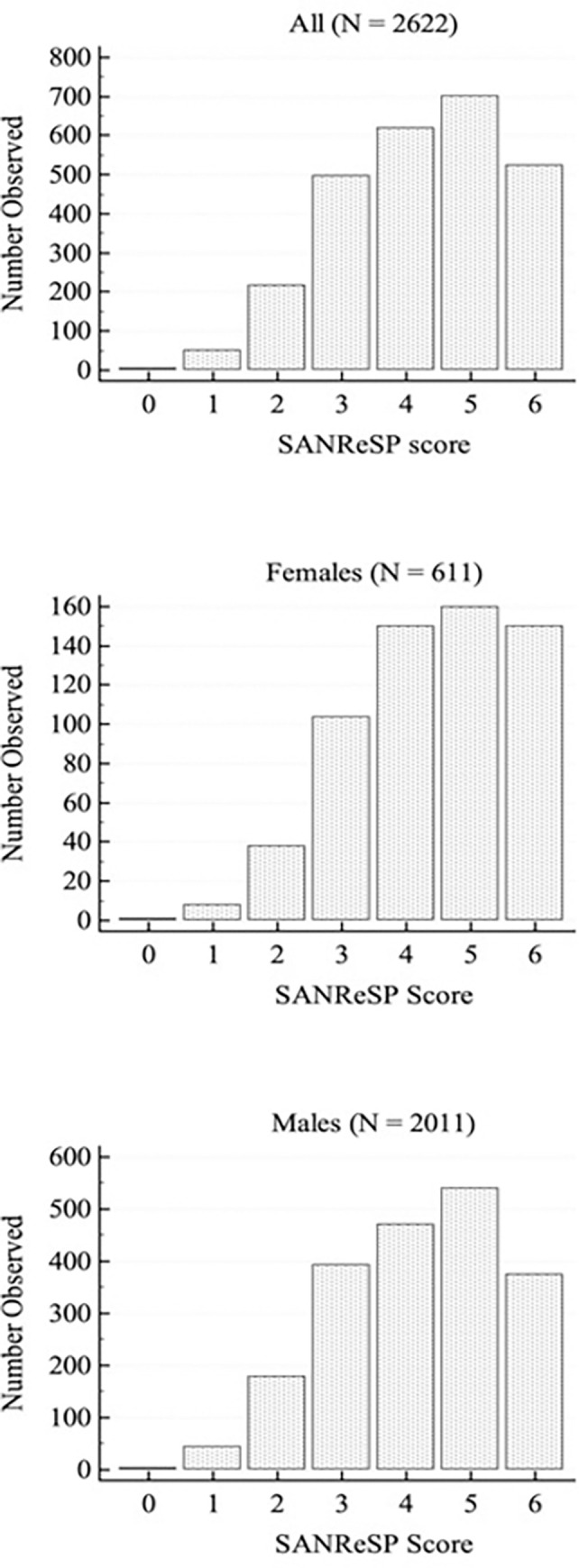
Distribution of patients according to their SANReSP score for males and females.

**Fig 3:**
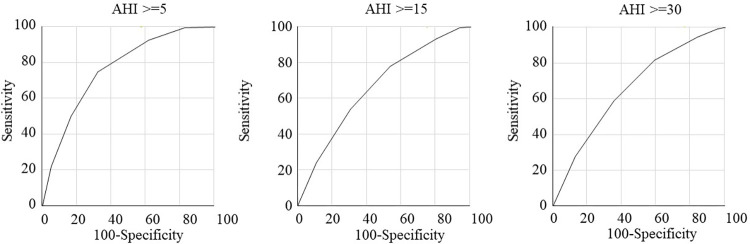
Receiver operating characteristic curves analysis of the SANReSP for all subjects.

**Fig 4:**
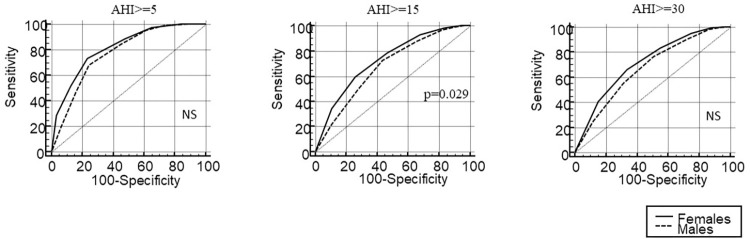
Receiver operating characteristic curves analysis of the SANReSP for males and females.

The median SANReSP score was 4 for men and 5 for women. In Table 2, we can see the positive responses for SANReSP, from the total population and between males and females. Fig 2 shows the distribution of the SANReSP scores for men and women. A score of 5 was most frequent in both sexes. Figs 3 and 4 show the ROC curves for all patients and men/women, respectively, at AHI severity levels of ≥ 5/h, ≥ 15/h, and ≥ 30/h. The comparison between the ROC curves of men and women were statistically different at AHI ≥ 15/h. Additionally, the AUCs for women were larger than that of men.

**Table 2:** Positive response of SANReSP.

	All	Males	Females
Snoring n (%)	2493 (95.0)	1922 (95.5)	571 (93.4)
Observed Apnea n (%)	2083 (79.4)	1642 (81.6)	441 (72.2)
Nocturia n (%)	1535 (58.5)	1120 (55.7)	415 (67.9)
Rest n (%)	2023 (77.1)	1523 (75.7)	500 (81.8)
Sleepy n (%)	1602 (61.0)	1200 (59.7)	402 (65.8)
High blood pressure n (%)	1386 (52.8)	1019 (50.7)	367 (60.0)

Data are presented in absolute numbers and percentages.

Evaluation of the questionnaire’s validity for AHI severity (≥ 5/h, ≥ 15/h, ≥ 30/h) is presented in Table 3. For AHI ≥ 5/h, the optimal SANReSP cut-off was >3 (sensitivity, 74.76%; specificity, 67.92%; accuracy, 74.07%; OR, 6.30; AUC, 0.76). For moderate–severe OSA (AHI ≥ 15/h), the optimal cut-off was >3 (sensitivity, 78.18%; specificity, 46.53%; accuracy, 68.27%; OR, 3.11; AUC, 0.66). For severe OSA (AHI ≥ 30/h), the optimal cut-off was >4 (sensitivity, 59.10%; specificity 64.73%; accuracy, 62.01%; OR, 2.67; AUC, 0.65).

**Table 3:** Predictive parameters for SANReSP questionnaire.

**All OSA, AHI≥ 5 All**		**Males**	**Females**
Sensitivity, %	74.76 (72.95–76.50)	72.55 (70.45–74.56)	82.59 (79.04–85.76)
Specificity, %	67.92 (61.94–73.50)	69.59 (62.11–76.38)	64.89 (54.36–74.46)
PPV, %	95.40 (94.56–96.11)	96.25 (95.33–96.99)	92.83 (90.74–94.47)
NPV, %	23.2 (21.4–25.2)	19.07 (17.23–21.6)	40.40 (34.79–46.27)
LR+	2.33 (1.95–2.78)	2.39 (1.90–3.00)	2.35 (1.78–3.11)
LR-	0.37 (0.33–0.41)	0.39 (0.35–0.45)	0.27 (0.21–0.34)
Accuracy, %	74.07 (72.34–75.73)	72.30 (70.29–74.25)	79.87 (76.47–82.98)
OR	6.30 (4.76–8.25)	6.12 (4.29–8.51)	8.70 (5.42–14.18)
Optimal cut-off	>3	>3	>3
Area under ROC curve	0.76 (0.75–0.78)	0.77(0.75–0.79)	0.81 (0.78–0.84)
**Moderate-severe OSA, AHI≥ 15**			
Sensitivity, %	78.18 (76.20–80.07)	76.13 (73.84–78.31)	63.61 (58.41–68.59)
Specificity, %	46.53 (43.04–50.01)	49.12 (44.94–53.31)	67.73 (61.56–73.47)
PPV, %	76.23 (74.97–77.45)	79.09 (77.64–80.47)	73.87 (69.93–77.47)
NPV, %	49.29 (46.44–52.14)	44.87 (41.82–47.97)	56.48 (52.49–60.39)
LR+	1.46 (1.37–1.57)	1.50 (1.37–1.63)	1.97 (1.62–2.40)
LR-	0.47 (0.42–0.53)	0.59 (0.43–0.55)	0.54 (0.46–0.3)
Accuracy, %	68.27 (66.45–70.05)	68.47 (66.39–70.50)	65.30 (61.38–69.08)
OR	3.11 (2.61–3.72)	3.07 (2.51–3.77)	3.66 (2.60–5.15)
Optimal cut-off	>3	>3	>4
Area under ROC curve	0.66 (0.64–0.68)	0.67 (0.65–0.69)	0.73 (0.69–0.76)
**Severe OSA, AHI≥ 30**			
Sensitivity, %	59.10 (56.33–61.82)	56.80 (53.72–59.84)	69.60 (63.17–75.52)
Specificity, %	64.73 (62.12–67.27)	66.43 (63.36–69.39)	60.42 (55.33–65.34)
PPV, %	60.93 (58.88–62.94)	64.30 (61.90–66.63)	50.97 (47.21–54.72)
NPV, %	62.97 (61.15–64.7)	59.09 (57.07–61.07)	77.08 (73.10–80.62)
LR+	1.68 (1.54–1.82)	1.69 (1.53–1.88)	1.76 (1.51–2.04)
LR-	0.63 (0.59–0.68)	0.65 (0.60–0.71)	0.50 (0.41–0.62)
Accuracy, %	62.01 (60.12–63.88)	61.46 (59.29–63.60)	63.83 (59.88–67.65)
OR	2.67 (2.26–3.10)	2.60 (2.17–3.11)	3.52 (2.46–4.95)
Optimal cut-off	>4	>4	>4
Area under ROC curve	0.66 (0.64–0.68)	0.68 (0.66–0.70)	0.70 (0.68–0.74)

Data are presented as average (95% confidence interval). AHI, apnea-hypopnea index; NPV, negative predictive value; PPV, positive predictive value; LR+, likelihood ratio of a positive test; LR-, likelihood ratio of a negative test; OR, odd ratio; ROC, receiver operating characteristic.

**Fig 5:**
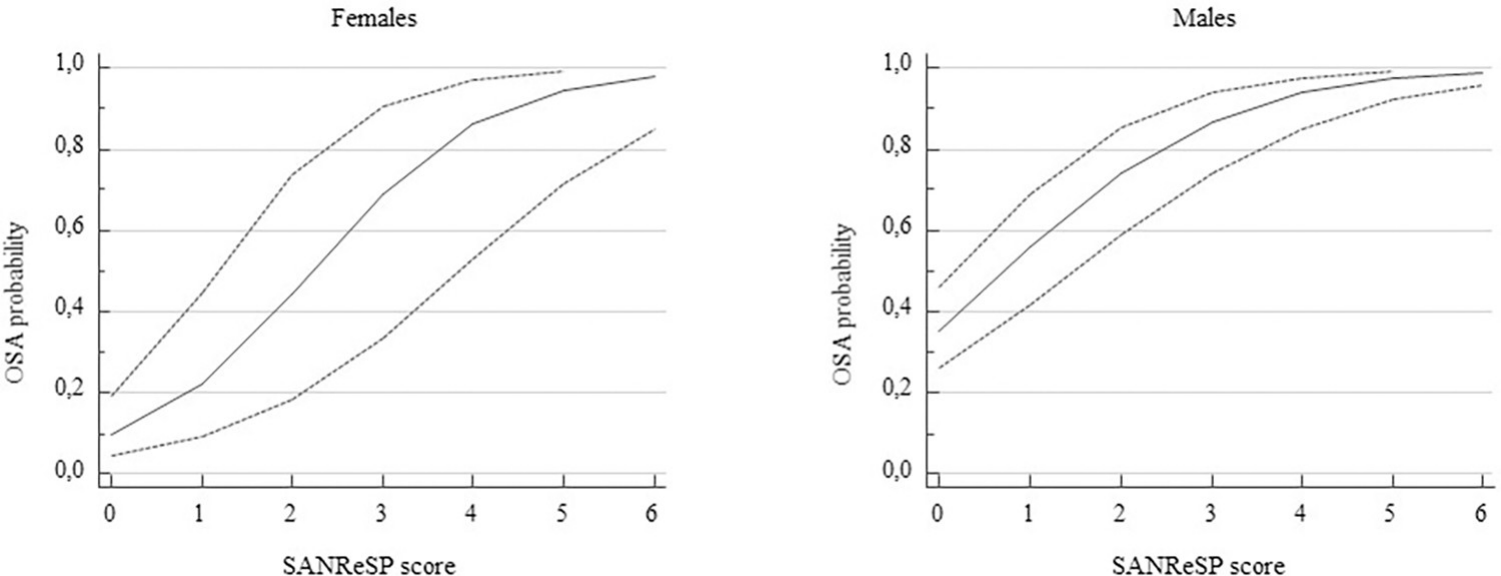
Predicted probabilities plot for AHI ≥5/h, with the corresponding SANReSP score. Dashed lines indicate the 95% confidence intervals.

Fig 5 shows the relationship between the predictive probability of sleep apnea (AHI ≥ 5/h) versus the SANReSP questionnaire score; the dashed line indicates the 95% CIs. The probability of OSA increased as the SANReSP score increased, with the same trend in both women and men. The probability of AHI ≥ 5/h was 86.8% in men and 68.7% in women for a score of 3, while it was 98.7% in men and 97.9% in women for a score of 6.

## Discussion

An ideal screening tool for detecting OSA in the general or sleep clinic populations should consist of simple questions combined with good sensitivity and specificity. In the general population, questionnaires with high sensitivity and LR- are generally desired, where-as in specific populations, questionnaires with high specificity are needed to promptly initiate appropriate therapeutic measures [15, 17]. The short questionnaires most commonly used to detect OSA are the BQ and SBQ along with the ESS for the subjective assessment of daytime sleepiness.

The BQ is a screening test designed for primary care administration, with 10 questions allocated in-to three categories. The first category includes five questions on snoring and cessation of breathing, the second category includes four questions on daytime sleepiness and fatigue, and the last category includes one question on systemic arterial hypertension and obesity. Patients with positive scores in two or more categories are defined as being at high risk for OSA [18]. A meta-analysis of 42 studies found sensitivity and specificity levels of 76% and 59% for mild OSA, 77% and 44% for moderate OSA, and 77% and 44% for severe OSA [9].

The SBQ is a simple OSA screening tool, partly related to symptoms (STOP) and physical measures (BANG).

The total score ranges from 0 to 8, with a score between 5 and 8 classified as high risk for OSA [12].

The SBQ, developed in the anaesthesiology field, has been validated in different contexts (different ethnic groups, general population, sleep clinic populations, commercial drivers, patients with cardiovascular factors) and it is now widely used to identify patients at high risk for OSA [19–21].

Different BMI thresholds for OSA have been suggested among ethnic groups. BMIs of 27.5 kg/m2 in Chinese and Indian populations and 35 kg/m2 in Malay and Caucasian populations predict moderate–severe OSA [22]. A meta-analysis of 11 sleep clinic studies with multiethnic populations (47–1426 participants) revealed that the SBQ predicted OSA (AHI ≥ 5) at a sensitivity and specificity ranging from 81% to 95% and 31% to 58%, respectively [23]. The advantage of the SANReSP questionnaire in a sleep clinic is in the speed and simplicity of the screening method and the absence of anthropometric parameters that allows patient interviews to be conducted using telephone or videoconferencing. One study evaluated the sensitivity and specificity of the SBQ among patients referred to a sleep disorder laboratory where physical measurements of BANG were reported by the patient rather than measured by the clinic staff. The use of self-reported values (height, weight, sex, age, and neck circumference), rather than measurements by sleep clinic staff, reduced the sensitivity of the SBQ [19]. A new four-item questionnaire, named GOAL (gender, obesity, age, and loud snoring), has been developed and validated [24].

The SANReSP questionnaire has the advantage of not including questions that require direct measurement of patient parameters. The convenience of a screening tool that does not require physical assessment increases its use in large-scale studies. The SANReSP questionnaire adds an important symptom of the clinical picture of OSA, namely nocturia. OSA is a frequent cause of nocturia, especially in younger individuals without urological disorders. The prevalence of nocturia in patients with OSA is 50–70% [25].

The ESS is used worldwide for the subjective evaluation of the propensity to fall asleep in specific daytime situations. It includes eight self-rated items, and each item is scored from 0 to 3. A score of >10 indicates significant daytime sleepiness. The ESS was initially proposed for the assessment of excessive daytime sleepiness (EDS) in narcolepsy, but it has subsequently been widely used for assessing EDS in OSA. Studies have shown that the ESS is a weak predictor of OSA, but it is useful when combined with other measures [26]. The ESS correlates only modestly with ‘objective’ measures of sleepiness, such as the sleep latency test and maintenance of wakefulness test, while it correlates very weakly with the severity of OSA defined by both AHI and nocturnal oxygen saturation.

### Strengths and limitations

The SANReSP questionnaire is a low-cost, easy, short, and self-administered questionnaire, which exclusively consists of clinical parameters and does not require patient measurements. Thus, it is excellent for screening patients with suspected OSA. The sample size of the study is among the largest sample sizes used to investigate the predictivity of OSA screening questionnaires. A limitation of the study is the use of home polygraphic studies for diagnostics rather than complete laboratory-based polysomnography. PSG is widely accepted as the gold standard test for diagnosis of OSA. However, within the appropriate context, the use of Level 3 sleep study is considered an acceptable method for diagnosing OSA by the American Academy of Sleep Medicine [27]. As reported by AASM Rules, Level 3 sleep study were performed after clinical evaluation. Additionally, sleep assessment was performed under the supervision of certified sleep medicine physician. Furthermore, the study has a selection bias since the results apply to a sleep clinic population, i.e. patients with a high probability of pre-test disease, which is not representative of the general adult population. Additionally, the sensitivity of this tool is moderate and some of the patients referred to sleep clinic can be missed. Furthermore, the questionnaire development was based on a literature review and questions were related to the most common clinical domain of OSA. No factor analysis was performed.

## Conclusion

In light of the profound impact of OSA on health and quality of life, it is essential that patients are adequately screened to receive the necessary medical care. Thus, a screening tool is necessary to stratify patients based on clinical symptoms and risk factors. The performance of the SANReSP questionnaire is similar to other widely used OSA screening questionnaires; moreover, the possibility of its use without the measurement of anthropometric parameters makes it a valid screening tool that can be administered using telephone or video-conferencing in sleep clinic populations. In conclusion, the SANReSP questionnaire is a short, easy-to-use, and self-administered screening tool for OSA. The questionnaire needs further investigation as a possible aid in the public health efforts to identify populations at risk of OSA, a frequently unrecognised sleep disorder with serious associated morbidity and mortality.

## Supporting information

S1 Appendix SANReSP questionnaire. (DOCX)
