# Supplementary material for: SANReSP: A new Italian questionnaire to screen patients for obstructive sleep apnea
Source: PLoS One. 2022 Oct 14;17(10):e0276217. doi: 10.1371/journal.pone.0276217 (PMC9565397; doi:10.1371/journal.pone.0276217)
Supplement: S1 Appendix — (DOCX) [file pone.0276217.s001.docx]

**Appendix A – SANReSP Questionnaire**

**1 Do they tell you that you snore? (Snore)**

**[Le dicono che russa?]**

**2 Do they tell you that sometimes you stop breathing or have sleep apnea? (Apnea)**

**[Le dicono che talvolta smette di respirare o ha apnee durante il sonno?]**

**3 Do you wake up during the night with an urge to urinate? (Nocturia)**

**[Si sveglia durante la notte con il bisogno urgente di urinare?]**

**4 Does it happens to you not being satisfied with how you slept? (Rest)**

**[Le capita di non essere soddisfatto/a di come ha dormito?]**

**5 Do you frequently feel the desire or need to sleep during the day except after lunch? (Sleepy)**

**[Sente frequentemente il desiderio o il bisogno di dormire durante il giorno eccetto dopo pranzo?]**

**6 Do you take medications for high blood pressure? (Pressure)**

**[Assume farmaci per la pressione arteriosa alta?]**
